# IL-27 Promotes Proliferation of Human Leukemic Cell Lines Through the MAPK/ERK Signaling Pathway and Suppresses Sensitivity to Chemotherapeutic Drugs

**DOI:** 10.1089/jir.2015.0091

**Published:** 2016-05-01

**Authors:** Haiyan Jia, Paula Dilger, Chris Bird, Meenu Wadhwa

**Affiliations:** Cytokines and Growth Factors Section, Biotherapeutics Group, National Institute for Biological Standards and Control, Potters Bar, United Kingdom.

## Abstract

IL-27 is a pleiotropic cytokine of the IL-6/IL-12 family with diverse biological functions. Previous *in vivo* studies have suggested the antitumor activities of IL-27 in animal models, whereas clinical observations indicate the link of IL-27 in tumor progression. IL-27 has recently been shown to cause inhibition of proliferation on primary leukemic cells from pediatric patients, but information on its role in human leukemic cell lines is limited. In the present study, we investigated the ability of IL-27 to regulate cell growth and survival of various human leukemic cell lines. Our results showed that in human leukemic cell lines coexpressing both IL-27R chains, IL-27Rα and gp130, IL-27 did not inhibit cell growth, but caused dose-dependent proliferation of the acute myeloid leukemic cell line, OCI-AML5, and the erythroleukemic cell lines, TF-1, UT-7, and UT-7/EPO. Consistent with this, IL-27 promoted cell survival and reduced TNF-α-induced apoptosis of the leukemic cell lines. IL-27 also decreased the responsiveness of the leukemic cells to chemotherapeutic drugs, cytarabine and daunorubicin. We observed that IL-27 induced the activation of STAT1/3 and ERK1/2 in the leukemic cells. Growth stimulation by IL-27 was suppressed by the specific MEK inhibitor, U0126, indicating that IL-27-induced cell proliferation is mainly mediated through the activation of the MAPK/ERK signaling pathway. The present study is the first demonstration of the proliferative and antichemotherapeutic properties of IL-27 in human leukemic cell lines, suggesting that IL-27 can play an unfavorable role in tumor growth and can be an important determinant in the chemoresponsiveness of certain subtypes of human leukemia.

## Introduction

Interleukin-27 is a heterodimeric cytokine of the IL-6/IL-12 family comprising the cytokine subunit p28 and the soluble cytokine receptor Epstein–Barr virus-induced gene 3 (EBI3) (Pflanz and others 2002). Mainly produced by activated antigen-presenting cells, including dendritic cells and macrophages upon exposure to physiological stimuli, IL-27 signals through a heterodimeric receptor consisting of 2 chains, the specific IL-27Rα (WSX-1 or TCCR) paired with the signal-transducing gp130 that is shared by the IL-6 family of cytokines (Pflanz and others 2004), and activates the signal transducer and activator of transcription (STAT) pathway. IL-27R components are expressed on a wide variety of immune, hematopoietic, endothelial, and epithelial cells, resulting in a variety of cellular targets and diverse functions for its ligand. IL-27 functions as a pleiotropic cytokine that is capable of modulating immune response, inflammation, hematopoiesis, and tumor growth (Hunter and Kastelein 2012; Adamopoulos and Pflanz 2013).

IL-27 was initially discovered as a cytokine that promotes CD4^+^ T-cell proliferation and the early stages of helper T (Th)1 cell differentiation (Pflanz and others 2002; Takeda and others 2003) and was later identified as an immunoregulator able to suppress Th1, Th2, and Th17 cell responses. The inhibitory activities of IL-27 include the abilities to antagonize T-cell production of the proinflammatory cytokine IL-2 (Villarino and others 2006; Owaki and others 2006) to induce production of the anti-inflammatory cytokine IL-10 by type 1 regulatory T cells (Awasthi and others 2007; Stumhofer and others 2008; Pot and others 2011) and to upregulate expression of the suppressive molecule, programmed death ligand 1 (PD-L1 or B7-H1), by dendritic cells and T cells (Karakhanova and others 2011; Hirahara and others 2012). Therefore, IL-27 plays a dual role in the regulation of inflammation by immunostimulatory or immunosuppressive functions on target cells (Yoshida and others 2009; Hunter and Kastelein 2012).

Since IL-27 was first reported as having antitumor activity in animal models of colon cancer and neuroblastoma in 2004 (Hisada and others 2004; Salcedo and others 2004), the tumor-suppressive ability of IL-27 has been verified in various murine tumor models, including solid tumors as well as hematological malignancies (Oniki and others 2006; Murugaiyan and Saha 2013; Liu and others 2013). IL-27 displays antitumor activity *in vivo* through multiple mechanisms, including antitumor immunity and antiangiogenesis activity, depending on the characteristics of tumor models. However, emerging studies indicate that this cytokine can also have some tumor-promoting properties through induction of IL-10 production (Awasthi and others 2007; Stumhofer and others 2008; Pot and others 2011) and PD-L1 expression (Karakhanova and others 2011; Hirahara and others 2012), suggesting that its antitumor immunity may be limited by the potent immunosuppression mediated by IL-10 and PD-L1. In support of these notions, accumulated evidence from clinical studies has shown that IL-27 and IL-27R are present in various tumor types from patient specimens, and significantly increased serum levels of IL-27 correlate with tumor growth and disease progression (Larousserie and others 2005, 2006; Diakowska and others 2013; Gonin and others 2013; Lu and others 2014).

While many studies have focused on *in vivo* animal models, fewer *in vitro* studies investigated the effects of IL-27 on human tumor cell biology, including human melanoma cell lines (Shimizu and others 2006; Yoshimoto and others 2008) and primary tumor cells from patients with leukemia (Canale and others 2011; Zorzoli and others 2012), and showed antiproliferative activity of IL-27. Interestingly, studies of the leukemic cells from a patient with acute myeloid leukemia (AML) have demonstrated that IL-27R possesses hematopoietic cell-transforming properties, suggesting its implication in myeloproliferative neoplasms (Pradhan and others 2007, 2010). However, the role of IL-27 in modulation of cellular growth and survival of human leukemic cell lines still remains undefined. Since leukemic cell lines have been widely used as models for the study of the mechanisms regulating cell proliferation, an understanding of the role of IL-27 in regulation of growth of various subtypes of human leukemic cells is of biological importance and clinical relevance.

Therefore, the primary aim of this study was to assess the role of IL-27 in regulation of human leukemic cell growth. We investigated the direct biological effects of IL-27 *in vitro* on different subtypes of human leukemic cell lines coexpressing both IL-27Rα and gp130 identified from a panel of leukemic cell lines. Results obtained from examining seven IL-27R-positive leukemic cell lines showed that IL-27 was unable to display antiproliferative effects, but promoted cell growth of several leukemic cell lines. We therefore undertook further experiments to enable a better understanding of the specific effects of IL-27 on cellular activities of the responsive leukemic cells and to explore the molecular mechanisms underlying such actions.

## Materials and Methods

### Reagents

Recombinant human IL-27, recombinant human TNF-α, recombinant human IL27Rα-Fc chimera, recombinant human gp130-Fc chimera, and functional blocking antibody against human gp130 were purchased from R & D Systems (Abingdon, United Kingdom). Cell culture media, cell dissociation solution, phorbol 12-myristate 13-acetate (PMA), U0126, wortmannin, cytarabine, and daunorubicin were purchased from Sigma-Aldrich (Dorset, United Kingdom). All other reagents used were of the purest grade available.

### Cell culture

Human tumor cell lines were obtained from in-house sources. Human AML cell lines, KG-1, HL-60, OCI-AML5, and U937, human erythroleukemic cell line, TF-1, and human acute T-cell leukemic cell line, Jurkat, were cultured in RPMI 1640 medium supplemented with 10% heat-inactivated fetal bovine serum (HIFBS). Human erythroleukemic cell lines, UT-7 and UT-7/EPO, were maintained in Iscove's DMEM supplemented with 10% HIFBS. Because OCI-AML5, TF-1, and UT-7 are growth factor-dependent cell lines (Kitamura and others 1989; Wang and others 1991; Komatsu and others 1991), granulocyte–macrophage colony-stimulating factor (GM-CSF) was added to the growth medium for OCI-AML5, TF-1, and UT-7 cell lines. Erythropoietin (EPO) was added to the growth medium for UT-7/EPO cell line, which is also a factor-dependent cell line and was derived from UT-7 (Komatsu and others 1993). Human melanoma cell line, A375, was cultured in DMEM supplemented with 5% HIFBS.

### Gene expression analysis

Total RNA was extracted from human tumor cells and treated with DNase I to remove any possible genomic DNA by using the RNeasy Mini Kit (Qiagen, Manchester, United Kingdom), according to the manufacturer's instructions. Single-stranded cDNA was reverse transcribed from RNA with oligo (dT)_15_ primers, MMLV reverse transcriptase, and RT reaction buffer (Promega, Southampton, United Kingdom). cDNA was amplified by PCR for 40 cycles in a 20 μL reaction mixture containing PCR buffer, 0.5 μM of each primer, 250 μM of each dNTP, 1.5 mM of MgCl_2_, and 1 U of GoTaq DNA polymerase (Promega). Each cycle of PCR included 1 min of denaturation at 94°C, 45 s of primer annealing at 57°C or 59°C, and 2.5 min of extension at 72°C, and the final extension was 10 min at 72°C using the Master Cycler System (Eppendorf, Stevenage, United Kingdom). Amplification of predicted fragments was verified by 2% agarose gel.

Forward and reverse oligonucleotide primer pairs for PCR were designed specifically to match the sequences of human IL-27Rα and human gp130 using Primer3 software and synthesized by Integrated DNA Technologies. The forward and reverse primer pairs for each gene were from 2 different exons spanning a large intron so that PCR products arising from cDNA could be distinguished from any possible contaminating genomic DNA. The primer pairs are listed in Table 1.

**Table 1.** Primer Sequences Used for RT-PCR

*Gene*	*Accession number*	*Primer sequence (5′ to 3′)*	*PCR product (bp)*
IL-27Rα	NM_004843.3	Forward: CGA GTT ACA CCT CCA GAG CC	200
		Reverse: GGG CGT TTG GCT TCA TTT GG	
gp130	NM_002184	Forward: GTA CAA CTC GTG TGG AAG AC	239
		Reverse: GGG TGA GTA GCT TGA AAG TC	

RT-PCR, reverse transcription polymerase chain reaction.

### Flow cytometric analysis of IL-27R expression

Cells were first treated with a human Fc receptor blocking reagent (R&D Systems). Cell surface expression of IL-27Rα or gp130 was detected using PE-conjugated anti-IL-27Rα monoclonal antibody or PE-conjugated anti-gp130 monoclonal antibody (R&D Systems). In parallel, cells were stained with the respective isotype-matched control antibody, mouse IgG_2B_-PE, or mouse IgG_1_-PE monoclonal antibody (R&D Systems). The cells were analyzed on FACSCanto II (BD Biosciences, Oxford, United Kingdom), counting 30,000 cells. Data were analyzed using FACSDiva (version 6) and FlowJo (version 10) software and expressed as median fluorescence intensity (MFI).

### Cell proliferation

Cells were washed twice and seeded in 96-well plates at densities ranging from 5 × 10^3^ to 5 × 10^4^ cells per well in 200 μL assay medium supplemented with 5% or 10% HIFBS in the absence or presence of increasing concentrations of IL-27. Following stimulation for 2 or 3 days, the cells were pulsed with ^3^H-thymidine (0.5 μCi [0.0185 MBq]; Perkin Elmer, Coventry, United Kingdom) for the last 4 h, and ^3^H-thymidine incorporation was measured by scintillation counting (2450 MicroBeta2 Counter; Perkin Elmer). The factor-dependent cell lines were examined in the assay medium deprived of GM-CSF or EPO.

For drug response experiments, OCI-AML5, TF-1, UT-7, and UT-7/EPO cells were washed twice and seeded in 96-well plates at densities ranging from 2 × 10^4^ to 5 × 10^4^ cells per well in 200 μL of the assay medium containing various concentrations of cytarabine or daunorubicin as indicated in the absence or presence of IL-27 at 200 ng/mL. After 2 or 3 days, the cells were pulsed with ^3^H-thymidine for the last 4 h and ^3^H-thymidine incorporation was measured by scintillation counting.

### Cell viability

Cells were washed twice and seeded in 96-well plates at densities ranging from 2 × 10^4^ to 8 × 10^4^ cells per well in 100 μL assay medium supplemented with 2% HIFBS containing increasing concentrations of IL-27. After 44 h of incubation, the MTS-based CellTiter 96^®^ AQueous One Solution (Promega) was added to the cultures and the cells were incubated for a further 4 h. The formazan products converted from MTS tetrazolium by mitochondrial dehydrogenase of metabolically active cells were then measured at A_490_ nm using a plate reader (Spectra Max; Molecular Devices, Wokingham, United Kingdom). The factor-dependent cell lines were examined in the assay medium deprived of GM-CSF or EPO.

For signaling inhibitor experiments, TF-1 cells were washed twice and seeded in 96-well plates at a density of 4 × 10^4^ cells per well in 100 μL assay medium supplemented with 2% HIFBS (deprived of GM-CSF) containing various concentrations of U0126 or wortmannin as indicated in the absence or presence of IL-27 at 200 ng/mL. After 44 h of incubation, the MTS-based CellTiter 96 AQueous One Solution (Promega) was added to the cultures and the cells were incubated for a further 4 h. The formazan products were measured.

### Annexin V binding

Analysis of apoptosis was carried out using the apoptosis detection kit (Invitrogen, Paisley, United Kingdom) according to the manufacturer's instructions. Cells were washed twice and seeded in 96-well plates at a density of 2 × 10^5^ cells per well in 200 μL serum-free assay medium supplemented with 0.1% BSA and deprived of GM-CSF or EPO in the absence or presence of TNF-α, IL-27, or TNF-α together with IL-27 for 4-h or 24-h treatment. The cells were then washed and stained with fluorescein isothiocyanate (FITC)-conjugated Annexin V and propidium iodide (PI). After staining, the cells were analyzed by flow cytometry using FACSCanto II (BD Biosciences) and data were analyzed using FACSDiva software. The Annexin V-positive (Annexin V+) cells were counted as apoptotic cells, including those present in the lower right quadrant (Annexin V+/PI negative [PI−]) and the upper right quadrant (Annexin V+/PI+), respectively.

### Caspase-Glo 3/7 assay

Caspase-3/7 activities in leukemic cells were detected using the Caspase-Glo 3/7 assay kit (Promega) according to the manufacturer's instructions. Cells were washed twice and seeded in 96-well plates at a density of 1.5 × 10^4^ cells per well in 100 μL serum-free assay medium supplemented with 0.1% BSA and deprived of GM-CSF or EPO in the absence or presence of TNF-α, IL-27, or TNF-α together with IL-27. After 2.5 h of treatment, an equal volume of Caspase-Glo 3/7 reagent was added to each well, mixed, and incubated in the dark for 1 h at room temperature. The resultant luminescence that is proportional to caspase-3/7 activity was measured using a luminometer (2450 MicroBeta2 Counter; Perkin Elmer).

### Signaling pathways

IL-27-induced phosphorylation and activation of signaling molecules were investigated by flow cytometric analysis of intracellular staining. Cells were washed and either left untreated or treated with IL-27 in serum-free assay medium supplemented with 0.1% BSA for 15 min at 37°C. The cells were fixed in BD™ Phosflow Fix Buffer I containing formaldehyde for 10 min at 37°C, permeabilized in BD Phosflow Perm Buffer III containing 90% methanol for 30 min on ice, and then incubated simultaneously with PE-conjugated anti-STAT1-pTyr701 and PerCP-Cy™5.5-conjugated anti-STAT3-pTyr705 antibodies (BD Biosciences) for 45 min on ice. The dual-stained cells were analyzed on FACSCanto II (BD Biosciences). Data were analyzed using FACSDiva (version 6) and FlowJo (version 10) software and expressed as MFI.

For ERK1/2 and AKT activation, cells were starved in serum-free medium for 24 h, and then either left untreated or treated with IL-27 in serum-free assay medium supplemented with 0.1% BSA for 10 min at 37°C. The cells were fixed, permeabilized, and stained by using PE-anti-ERK1/2-pThr202/pTyr204 and Alexa Fluor 488-anti-AKT-pSer473 monoclonal antibodies of FlowCellect Dual Detection Kit (Merck Millipore, Watford, United Kingdom), according to the manufacturer's instructions.

### Binding kinetics

Surface plasmon resonance (SPR) measurements of binding kinetics of IL-27 with IL-27Rα-Fc or gp130-Fc and anti-gp130 antibody (Ab) with gp130-Fc were performed using a BIAcore T100 instrument (GE Healthcare, Little Chalfont, United Kingdom) at 25°C. Mouse anti-human IgG-Fc-specific antibodies were covalently coupled to a BIAcore CM5 sensor chip by amine coupling chemistry according to the manufacturer's instructions. Kinetic experiments were carried out using the multicycle mode. IL-27-Rα-Fc or gp130-Fc in running buffer (HBS-EP+) was captured onto the immobilized anti-human IgG-Fc antibodies of the chip surface, followed by sequential injections of IL-27 or anti-gp130 Ab at increasing concentrations over both the receptor chain-captured and the reference (nonreceptor chain-captured) surfaces at a flow rate of 30 μL/min. After each run, the sensor chip was regenerated by injection of 3 M magnesium chloride. The binding sensorgrams were double referenced before global fitting of the concentration series as both blank running buffer (no IL-27 or anti-gp130 Ab) and blank capture surface (no captured receptor chain) were used as references for background subtraction. Association and dissociation rate constants (*k_a_* and *k_d_*) were obtained by analyzing and fitting the primary sensorgram data according to the 1:1 Langmuir binding model using the BIAevaluation T100 software (version 2.0.3). Equilibrium affinity constant (*K_D_*) was derived from the kinetic parameters (*K_D_* = *k_d_*/*k_a_*).

### Statistical analysis

Data were analyzed using GraphPad Prism (version 5.0) statistical packages. Comparisons of 2 sets of continuous variables were performed using Student's *t* test or *t* test with Welch's correction where appropriate. Differences among 3 or 4 concentrations of compounds were evaluated using the one-way analysis of variance (ANOVA) with Bonferroni's multiple comparison tests. Differences between 2 treatment groups at various concentrations were analyzed using the 2-way ANOVA with Bonferroni's post-tests. Values represent mean ± SEM determined from the results of 3 independent experiments, each performed in duplicates or triplicates unless where stated. A value of *P* < 0.05 was taken as statistically significant.

## Results

### Identification of IL-27R-positive tumor cell lines

**FIG. 1.**
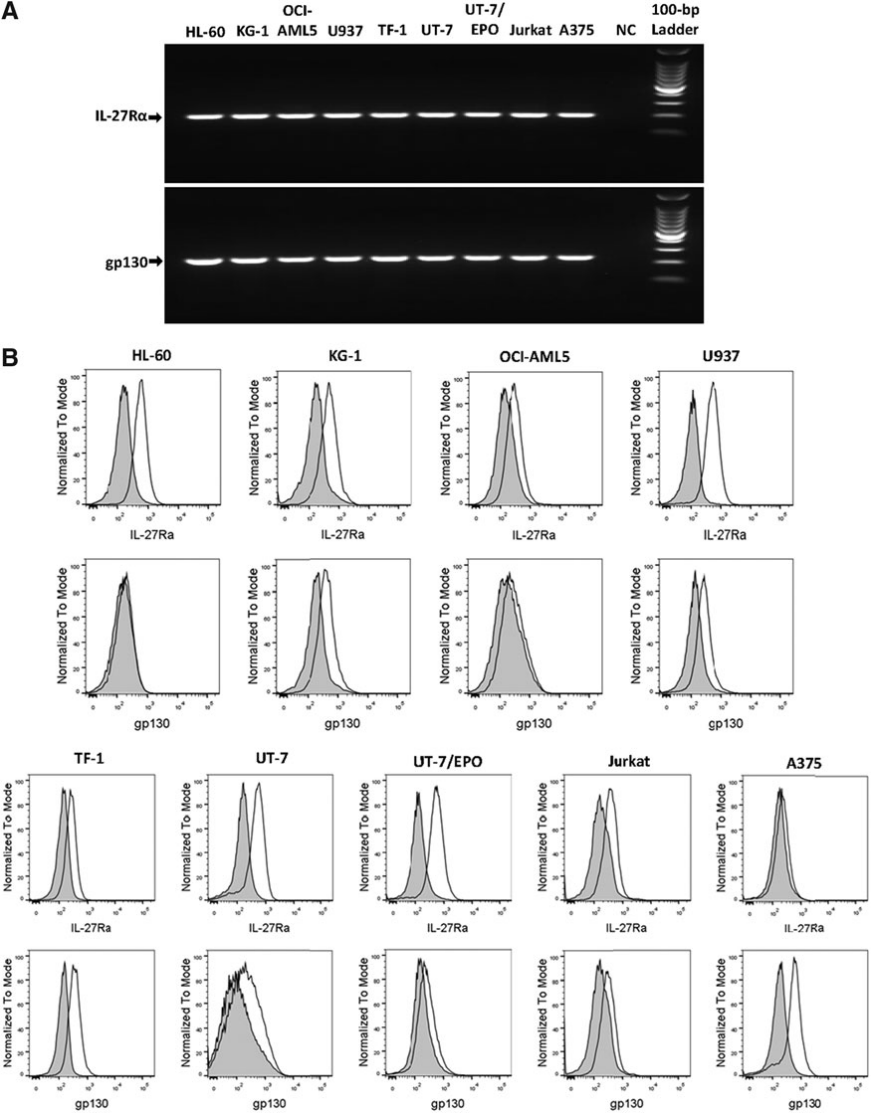
Expression of the IL-27R complex on human tumor cell lines. **(A)** mRNA expression of IL-27Rα and gp130 in 9 human tumor cell lines as indicated was analyzed by RT-PCR using specific primers. NC represents a negative control in which cDNA was replaced by H_2_O. The PCR products were separated by 2% agarose gel electrophoresis and the molecular weights were determined using the 100-bp DNA ladder. **(B)** Cell surface expression of IL-27Rα and gp130 on 9 human tumor cell lines as indicated was detected by flow cytometry using PE-conjugated specific antibodies. *Shaded* histograms are isotype-matched mAb staining. *Unshaded* histograms are IL-27Rα and gp130 staining, respectively. RT-PCR, reverse transcription polymerase chain reaction.

We first examined the expression of both chains, IL-27Rα and gp130, of IL-27R on a panel of nine tumor cell lines, including 4 AML cell lines (HL-60, KG-1, OCI-AML5, U937), 3 erythroleukemic cell lines (TF-1, UT-7, UT-7/EPO), an acute T-cell leukemic cell line (Jurkat), and a melanoma cell line (A375) at both mRNA and cell surface levels to identify IL-27R-positive tumor cell lines. Reverse transcription polymerase chain reaction (RT-PCR) analysis of the gene expression of the IL-27 receptor complex revealed bands of predicted sizes for both IL-27Rα and gp130 chains in the panel of cell lines (Fig. 1A and Table 1). The results confirm that both receptor components are constitutively coexpressed at the mRNA level in all tumor cell lines examined. Consistent with mRNA expression, flow cytometric analysis of the cell surface expression of the IL-27R complex showed that eight leukemic cell lines constitutively expressed IL-27Rα with MFI ranging from 1.8- to 4.5-fold higher than the isotype-matched control (Fig. 1B). However, gp130 was expressed on the cell surface of seven leukemic cell lines, except for HL-60, with MFI ranging from 1.5- to 2.4-fold higher than the isotype-matched control. For the melanoma cell line, A375, IL-27Rα was minimally detected, while gp130 was abundantly present at the surface level. Therefore, seven leukemic cell lines showed the presence of both IL-27 chains, suggesting these cell lines may respond to IL-27 modulation in their cellular activities.

### Effects of IL-27 on proliferation and viability of leukemic cell lines

**FIG. 2.**
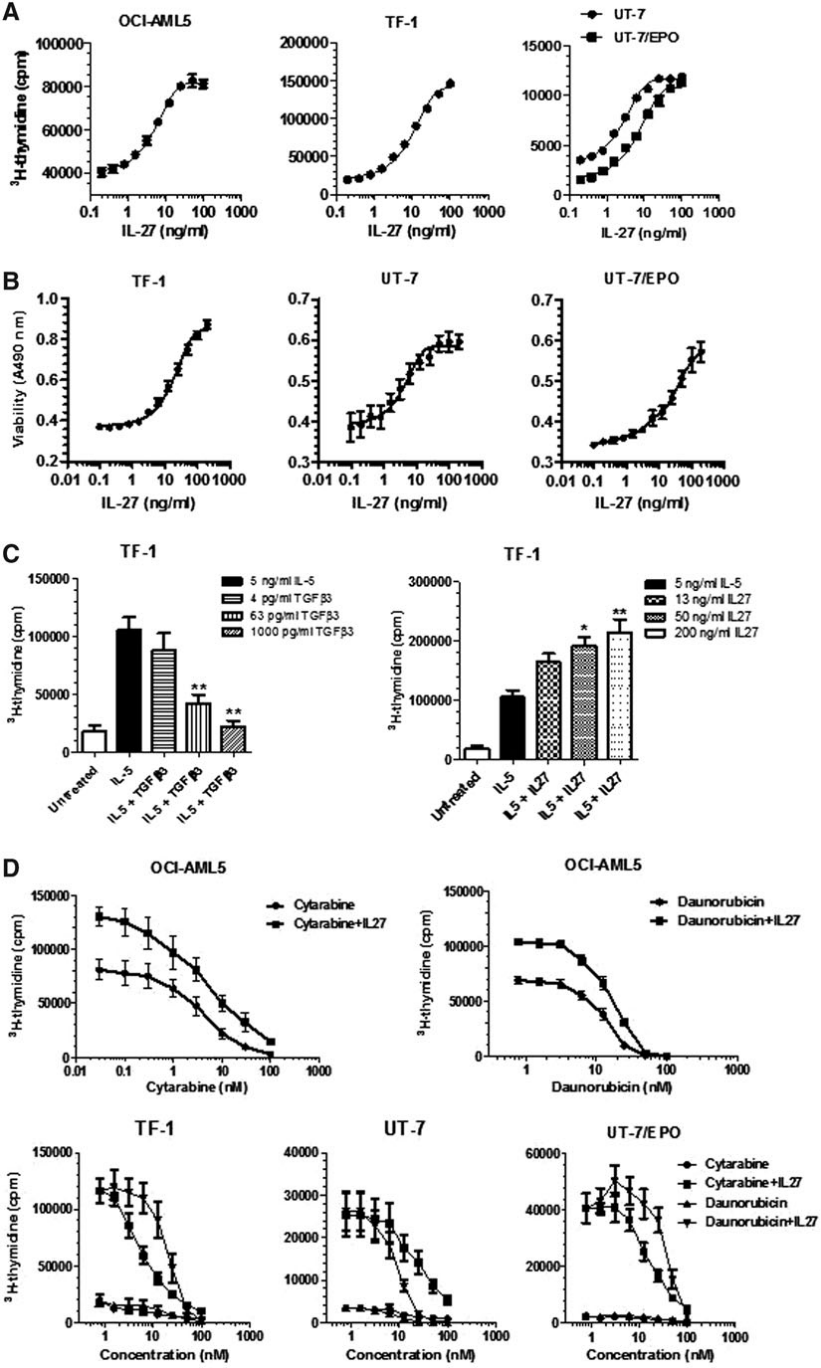
Proliferation stimulation and desensitization of human leukemic cells to the chemotherapeutic agents by IL-27. **(A)** Human leukemic cell lines were deprived of GM-CSF or EPO and cultured in 5% or 10% serum assay medium containing IL-27 at increasing concentrations. Dose–response relationships of IL-27 stimulation to cell proliferation were measured by ^3^H-thymidine incorporation after 2- or 3-day incubation. **(B)** Human leukemic cell lines were deprived of GM-CSF or EPO and cultured in 2% serum assay medium containing IL-27 at increasing concentrations. Dose–response relationships of IL-27 treatment to cell viability were measured by formazan production after 48 h of incubation. **(C)** TF-1 cells were deprived of GM-CSF and cultured in 5% serum assay medium containing IL-5 in the absence or presence of either TGF-β3 or IL-27 at indicated concentrations. Cell proliferation was measured by ^3^H-thymidine incorporation after 2-day incubation. **P < 0.01 for IL-5 plus TGF-β3 versus IL-5 alone. *P < 0.05 and **P < 0.01 for IL-5 plus IL-27 versus IL-5 alone. **(D)** OCI-AML5, TF-1, UT-7, and UT-7/EPO cells were deprived of GM-CSF or EPO and cultured in 5% or 10% serum assay medium containing cytarabine or daunorubicin at the indicated concentrations in the absence or presence of 200 ng/mL IL-27. Cell proliferation was measured by ^3^H-thymidine incorporation after 2-day incubation.

Next, we tested the ability of IL-27 *in vitro* to see if it could directly affect proliferation of leukemic cell lines, especially those which were IL-27Rα/gp130 double positive. The cells were incubated in the absence or presence of increasing concentrations of IL-27 in 10% serum assay medium for 2 or 3 days, followed by determination of ^3^H-thymidine incorporation. Results showed that IL-27 treatment caused a dose-dependent increase in cell proliferation of 4 leukemic cell lines, including the AML cell line, OCI-AML5, and the erythroleukemic cell lines, TF-1, UT-7, and UT-7/EPO (Fig. 2A). Unexpectedly, treatment with IL-27 at concentrations up to 200 ng/mL had no effects on cell growth of 3 other leukemic cell lines, the AML cell lines, KG-1 and U937, and the acute T-cell leukemic cell line, Jurkat, despite the surface expression of the full IL-27R complex (data not shown). As expected, the AML cell line, HL-60, and the melanoma cell line, A375, with little expression at cell surface of either gp130 or IL-27R did not respond to IL-27 stimulation (data not shown). Notably, the responsive leukemic cells are factor-dependent cell lines and require GM-CSF or EPO for growth maintenance as described in the Materials and Methods section. However, all experiments were performed in the assay medium without GM-CSF or EPO.

Having observed the growth stimulation of IL-27 in several leukemic cell lines, we then investigated the effects of IL-27 on survival of leukemic cell lines by seeding cells in 2% serum assay medium in the absence or presence of IL-27 at increasing concentrations for 48 h and measuring the mitochondrial dehydrogenase activity of viable cells. As for proliferation, IL-27 treatment promoted cell survival of 3 erythroleukemic cell lines, TF-1, UT-7, and UT-7/EPO, in a dose-dependent manner (Fig. 2B), but had a weak effect on OCI-AML5 cells (data not shown). Again, we were unable to find any effect of IL-27 on cell viability of the factor-independent leukemic cell lines, including KG-1, U937, HL-60, and Jurkat cells (data not shown).

It has previously been shown that IL-5 could stimulate proliferation of the erythroleukemic cell line, TF-1, and this effect could be inhibited by TGF-β (Randall and others 1993). To further assess any potential interference by IL-27, as seen in TGF-β, we carried out additional experiments to investigate the ability of IL-27 to affect cell proliferation in response to IL-5 stimulation. As expected in TF-1 cells, IL-5 treatment upregulated cell proliferation and this effect was inhibited by TGF-β3 (Fig. 2C). In striking contrast, TF-1 cell proliferation in response to IL-5 was augmented by the presence of IL-27 in a dose-dependent manner. The combined treatment with IL-5 and IL-27 resulting in an additive effect on cell proliferation is consistent with the growth-promoting activity of IL-27 on leukemic cells.

### Suppression of chemosensitivity in leukemic cell lines by IL-27

We further investigated whether IL-27 can modulate chemoresponse of leukemic cells as the assessment of disease response to induction chemotherapy is clinically important in AML. Treatment of OCI-AML5 cells with a chemotherapeutic agent, cytarabine, which is clinically used as frontline treatment for patients with AML, for 2 days caused dose-dependent inhibition of cell proliferation. However, the cytotoxic potency of cytarabine was reduced in the presence of IL-27 compared with the single agent cytarabine (Fig. 2D). More experiments were performed on the drug sensitivity using daunorubicin, which is another frequently used chemodrug for treating patients with AML. As shown in Fig. 2D, the cytotoxic activity of daunorubicin on OCI-AML5 cell growth was concentration dependent and exhibited similar inhibitory activity as cytarabine. Addition of IL-27 attenuated the cytotoxic effect of daunorubicin on OCI-AML5 cells compared with the chemodrug alone.

The antichemotherapeutic activity of IL-27 was further evaluated in the other 3 IL-27-responsive erythroleukemic cell lines, TF-1, UT-7, and UT-7/EPO. Clinically, cytarabine and daunorubicin are also most often used for treating patients with erythroleukemia. Both chemoagents revealed similar anticancer efficacy in TF-1, UT-7, and UT-7/EPO cell lines (Fig. 2D). However, the cytotoxic effect was dramatically decreased by combining IL-27 treatment with either cytarabine or daunorubicin compared with the drug alone, and IL-27 revealed greater antichemotherapeutic effect in the erythroleukemic cell lines than in the AML cell line (Fig. 2D). These results showed that IL-27 reproducibly decreased chemosensitivity of the erythroleukemic cell lines.

### Effects of IL-27 on apoptosis of leukemic cell lines

**FIG. 3.**
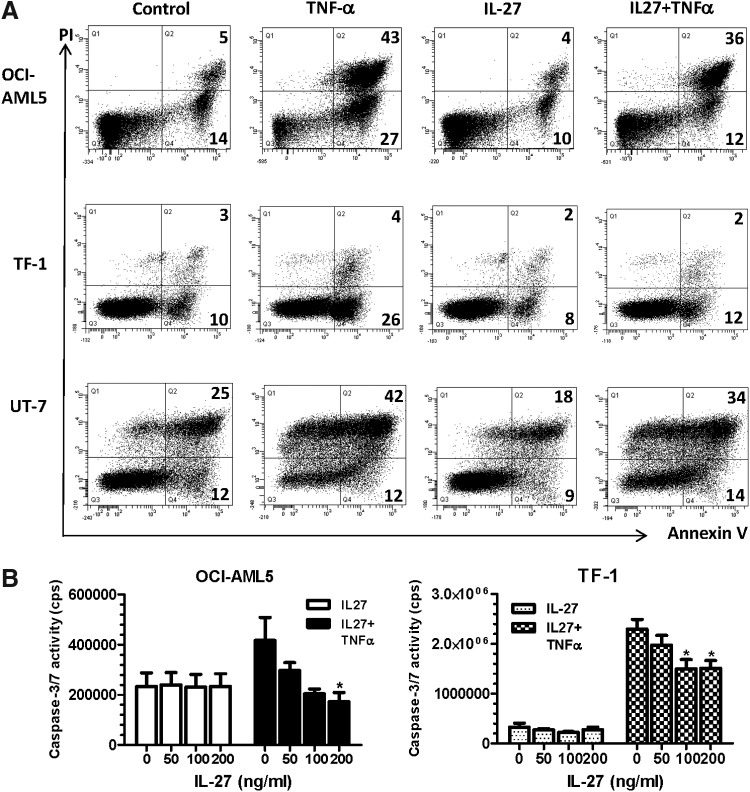
Inhibition of TNF-α-induced apoptosis by IL-27. **(A)** Leukemic cell lines were deprived of GM-CSF and cultured in serum-free assay medium containing 0.1% BSA in the absence or presence of 10 ng/mL TNF-α, 200 ng/mL IL-27, or 10 ng/mL TNF-α together with 200 ng/mL IL-27 for 4 h and 24 h. Following dual-staining with Annexin V-FITC and PI, percentages of apoptotic cells were determined by flow cytometric analysis of Annexin V+ cells. The Annexin V+ cells at early and late stages of apoptosis are shown as percentages in the *lower right* quadrant (Q4:% Annexin V+/PI−) and the *upper right* quadrant (Q2:% Annexin V+/PI+), respectively. Annexin V-FITC versus PI-PE plots with quadrant gates shown are representative of 3 independent experiments yielding very similar results. **(B)** OCI-AML5 and TF-1 cells were deprived of GM-CSF and cultured in serum-free assay medium containing 0.1% BSA in the absence or presence of 5 ng/mL TNF-α, IL-27 at indicated concentrations, or 5 ng/mL TNF-α together with IL-27 for 2.5 h. At the end of the treatment, the cells were lysed in Caspase-Glo 3/7 substrate and caspase-3/7 activities were assessed by the Caspase-Glo assay. *P < 0.05 for TNF-α plus IL-27 versus TNF-α alone.

The ability of IL-27 to influence apoptosis of the leukemic cell lines was also evaluated. Cells were untreated or treated with IL-27, followed by staining with FITC-Annexin V and PI. Annexin V binding to externalized phosphatidylserine is one hallmark of apoptosis, and PI, which stains DNA, is a marker for permeabilized dead cells at late-stage apoptosis. Annexin V+ cells, including both early (Annexin V+/PI−) and late (Annexin V+/PI+) apoptotic cell populations, were quantified by flow cytometry. TNF-α was used to serve as a positive control for 4-h and 24-h apoptosis induction and the leukemic cell responses varied. As shown in Fig. 3A, OCI-AML5 cells responded to TNF-α only after 24 h, but not 4-h induction, with a significant increase in Annexin V+ cells (70%) compared with the untreated control (19%). In contrast, IL-27-treated cells showed 14% of Annexin V+ cells, which was slightly lower compared with the untreated control. Cotreatment of cells with TNF-α and IL-27 decreased Annexin V+ cells to 48%, which was much lower than that seen with TNF-α alone (70%). The erythroleukemic cell line, TF-1, was responsive to TNF-α after 4 h of treatment, whereas UT-7 responded to TNF-α only after 24-h apoptosis induction (Fig. 3A). TNF-α-treated cells showed a marked increase in Annexin V+ cell populations compared with the untreated controls, 30% versus 13% in TF-1 and 54% versus 37% in UT-7, respectively. IL-27 treatment decreased Annexin V+ cells in TF-1 (10%) and UT-7 (27%) than those (13% in TF-1 and 37% in UT-7) of the respective untreated controls. TF-1 and UT-7 cells, in the presence of IL-27, revealed 14% and 48% of Annexin V+ populations in response to TNF-α treatment, which were again lower than those treated with sole TNF-α. However, UT-7/EPO cells were resistant to TNF-induced apoptosis even after 24 h of treatment (data not shown). These data suggest that IL-27 decreased the apoptotic response of the leukemic cells following exposure to TNF-α.

The role of IL-27 in leukemic cell apoptosis was further examined by assessing the activities of effector caspases 3 and 7, which cause apoptosis by cleaving cellular proteins (Thornberry and Lazebnik 1998). The leukemic cells were treated with the cytokine at various concentrations for 2.5 h of treatment, and the activity of caspases 3 and 7 was measured by determination of the cleavage of its substrate, the tetrapeptide sequence DEVD. Consistent with results of Annexin V binding, TNF-α increased caspase-3/7 activity of both OCI-AML5 and TF-1 cells. Addition of IL-27 caused concentration-dependent inhibition of TNF-induced caspase-3/7 activity in both cell lines, while IL-27 alone had no effect (Fig. 3B).

### Phosphorylation and activation of STAT pathway by IL-27

**FIG. 4.**
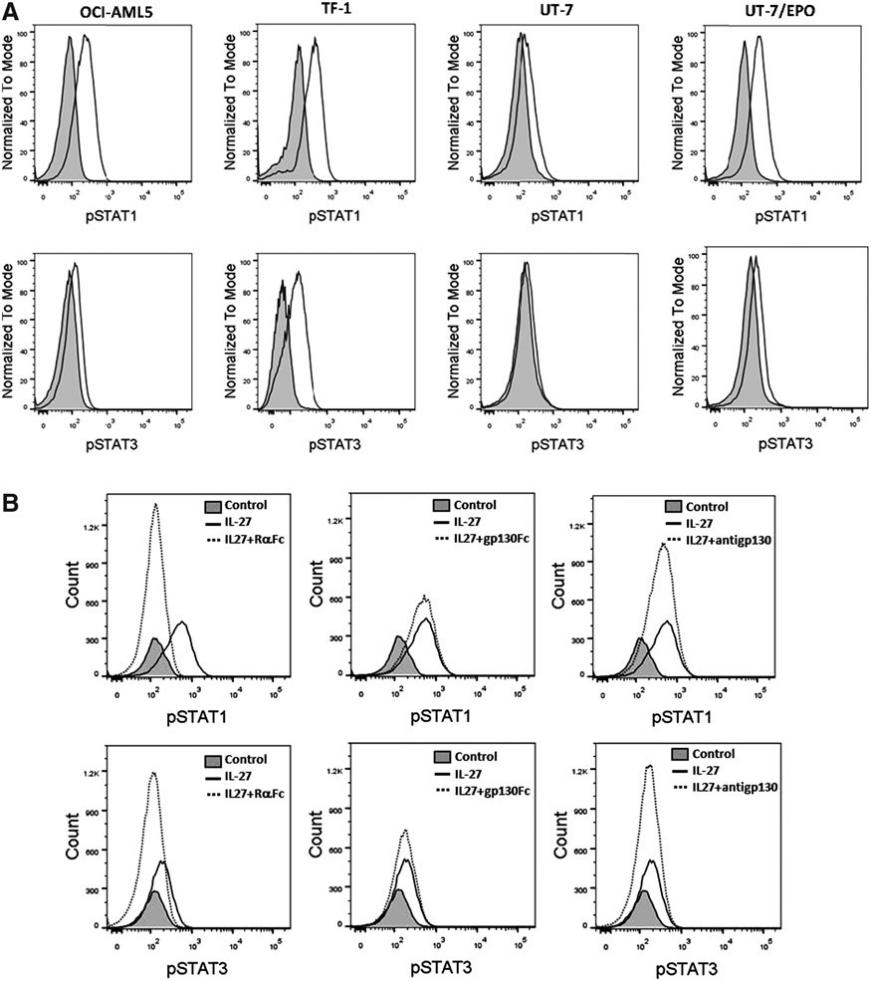

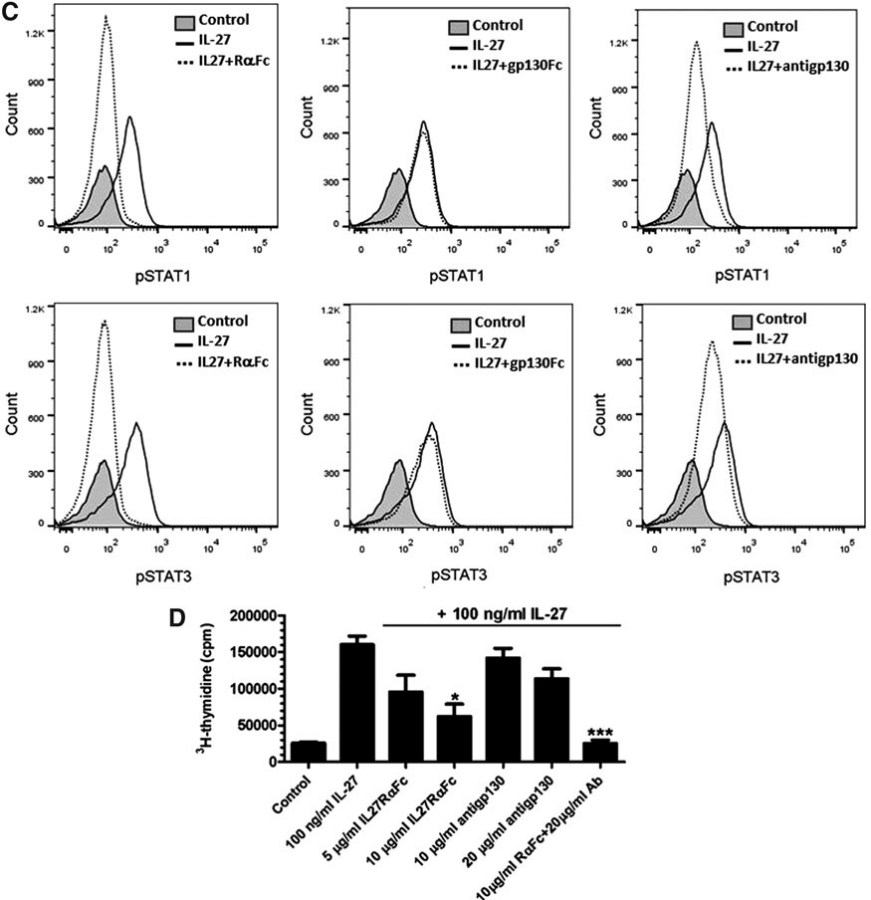
IL-27-activated STAT pathway was mediated through the receptor complex. **(A)** Intracellular phosphorylation of STAT1 and STAT3 activated by IL-27 was detected by flow cytometry on leukemic cell lines as indicated. *Shaded* histograms are untreated controls. *Unshaded* histograms are STAT1-pTyr701 (*top*) and STAT3-pTyr705 (*bottom*) staining, respectively. **(B, C)** IL-27 was preincubated for 15 min with either IL-27Rα-Fc or gp130-Fc before addition to OCI-AML5 **(B)** and TF-1 **(C)** cells for 15-min treatment. OCI-AML5 **(B)** and TF-1 **(C)** cells were pretreated with anti-gp130 Ab for 15 min, followed by IL-27 stimulation. Intracellular phosphorylation of STAT1 and STAT3 was analyzed by flow cytometry. **(D)** TF-1 cells were deprived of GM-CSF in 5% serum assay medium containing IL-27 in the absence or presence of either IL-27Rα-Fc or anti-gp130 Ab at indicated concentrations. Cell proliferation was measured by ^3^H-thymidine incorporation after 2-day incubation. *P < 0.05 for IL-27 plus IL-27Rα-Fc versus IL-27 alone. ***P < 0.001 for IL-27 plus both IL-27Rα-Fc and anti-gp130 Ab versus IL-27 alone.

To gain a better understanding of the cellular events and molecular mechanisms underlying IL-27 actions, we investigated the ability of the cytokine to induce activation of specific signaling molecules downstream of IL-27R in IL-27-responsive leukemic cell lines. The STAT1 and STAT3 pathways were analyzed since they have been reported to be involved in the IL-27 signaling pathway in immune cells and myeloid cells (Takeda and others 2003; Hibbert and others 2003; Pflanz and others 2004; Kamiya and others 2004) and implicated in IL-27R-mediated transformation of hematopoietic cells (Pradhan and others 2007). The activation of STAT1 and STAT3 occurs through tyrosine phosphorylation at Tyr701 and Tyr705, respectively. Therefore, the intracellular phosphorylation of STAT1-Tyr701 and STAT3-Tyr705 was examined by flow cytometry. IL-27 treatment induced a strong tyrosine phosphorylation of STAT1 in all responsive cell lines, OCI-AML5, TF-1, UT-7, and UT-7/EPO, with MFI of 2.8-, 2.6-, 1.6-, and 3.8-fold higher, respectively, than untreated controls. IL-27 also induced STAT3 phosphorylation in OCI-AML5, TF-1, and UT-7/EPO cells with MFI of 1.5-, 2.4-, and 1.5-fold higher, respectively, than untreated controls, but had little effect on STAT3 phosphorylation in UT-7 cells (Fig. 4A).

The specificity of the IL-27/IL-27R system in activation of STAT1 and STAT3 was also demonstrated by using a neutralizing anti-gp130 Ab and an IL-27Rα-Fc fusion protein because a neutralizing anti-IL-27Rα Ab is commercially unavailable. We either preincubated IL-27 at 100 ng/mL with the IL-27Rα-Fc fusion protein before cell stimulation or pretreated cells with the neutralizing anti-gp130 Ab, followed by IL-27 stimulation. IL-27-induced activation of both STAT1 and STAT3 was abolished by the IL-27Rα-Fc fusion protein and diminished by the neutralizing anti-gp130 Ab in OCI-AML5 (Fig. 4B) and TF-1 cells (Fig. 4C). In addition, the effect of the gp130-Fc fusion protein on IL-27-induced STAT phosphorylation was evaluated. Consistent with a previous report (Scheller and others 2005), incubation of the fusion protein with IL-27 before cell treatment produced little effect on IL-27-stimulated activation of both STAT1 and STAT3. The role of either IL-27Rα or gp130 in IL-27-mediated cellular responses was proved by inhibition of IL-27-induced proliferation of TF-1 cells with the IL-27Rα-Fc fusion protein or anti-gp130 Ab. The IL-27Rα-Fc fusion protein caused a dose-dependent inhibition of IL-27-mediated proliferation of TF-1 cells, while the neutralizing Ab against gp130 showed a weak reduction in IL-27-induced cell proliferation (Fig. 4D). Moreover, the combined pretreatment of anti-gp130 Ab with the IL-27Rα-Fc fusion protein completely abolished IL-27-induced cell proliferation (Fig. 4D). Notably, the IL-27R-Fc fusion protein showed a stronger blocking effect than the anti-gp130 Ab in TF-1 cells.

### Differential interaction of IL-27 with its each receptor chain

**FIG. 5.**
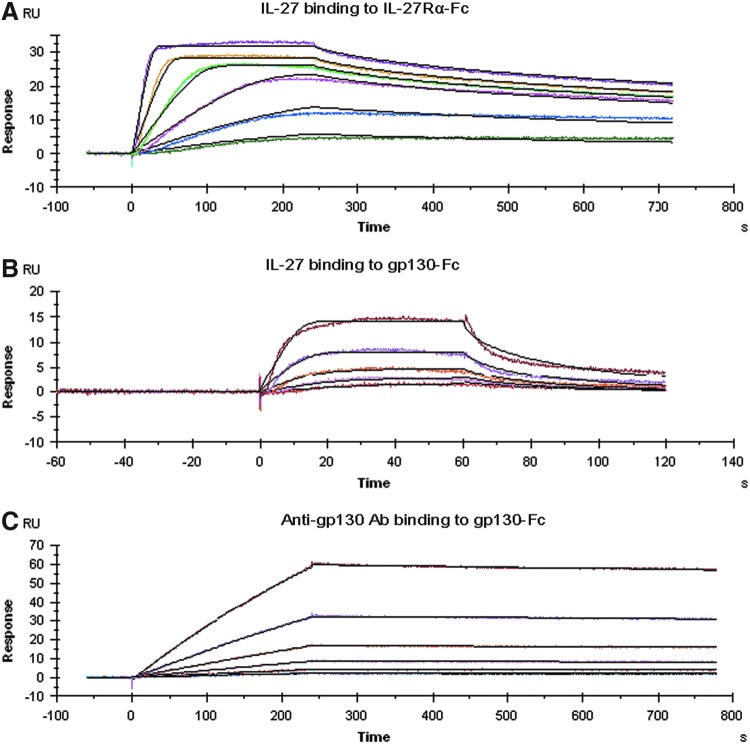
Kinetic binding interactions of IL-27 with each receptor chain and of anti-gp130 Ab with gp130. SPR sensorgrams were obtained from injections of IL-27 at concentrations of 0.25, 0.5, 1, 2, 4, and 8 nM over IL-27Rα-Fc captured surface **(A)**, human IL-27 at concentrations of 2, 4, 8, 16, and 32 nM over gp130-Fc captured surface **(B)**, and of anti-gp130 antibody at concentrations of 6.25, 12.5, 25, 50, 100, and 200 nM over gp130-Fc captured surface **(C)**. Experimental data were globally fitted with a 1:1 Langmuir binding model for the determination of kinetic constants. Each sensorgram shown is representative of 3 independent experiments. Color images available online at www.liebertpub.com/jir

To understand the reason for the disparate inhibition of IL-27-induced STAT1/3 signaling and cell proliferation by IL-27Rα and gp130 blockade, the binding kinetics of IL-27 interacting with each receptor chain were evaluated using SPR. The binding of IL-27 to the captured IL-27Rα-Fc is shown in Fig. 5A. Global fitting of the resulting sensorgrams to the parameters yielded a *k*_a_ of 9.1 × 10^7^ M^−1^ s^−1^, a *k*_d_ of 4.1 × 10^−3^ s^−1^, and a derived *K*_D_ of 46 pM, suggesting a slow disassociation rate, resulting in very high binding affinity (Table 2). Interestingly, we observed that IL-27 was also able to bind to gp130-Fc, in the absence of IL-27Rα, with a *k*_a_ of 1.7 × 10^11^ M^−1^ s^−1^, a *k*_d_ of 4.6 × 10^2^ s^−1^, and a derived *K*_D_ of 2.7 nM (Fig. 5B and Table 2), which revealed a 59-fold lower binding affinity than that of IL-27 binding to IL-27Rα. Both on and off rate constants for gp130-Fc binding to IL-27 were much faster than those for IL-27Rα-Fc interacting with IL-27. In addition, the kinetic parameters for the binding of the neutralizing Ab to gp130 were a *k*_a_ of 1.3 × 10^4^ M^−1^ s^−1^, a *k*_d_ of 1.1 × 10^−4^ s^−1^, and a derived *K*_D_ of 8.8 nM, which showed low binding affinity due to slow association and disassociation rates (Fig. 5C and Table 2). This indicated different binding kinetics of anti-gp130 Ab for gp130 as much lower affinity was seen compared with IL-27 binding to IL-27Rα-Fc. Therefore, the low binding affinity of the neutralizing anti-gp130 Ab may explain, at least in part, its relatively weak effect on blocking IL-27 signaling and function.

**Table 2.** Binding Kinetic Rate Constants and Affinities of IL-27 Interacting with Either IL-27Rα-Fc or gp130-Fc and of Anti-gp130 Ab Interacting with gp130-Fc

*Interaction*	*k_a_ (M*^−1^*s*^−1^*)*	*k_d_ (s*^−1^*)*	*K_D_ (M)*
IL-27 to IL-27Rα-Fc	9.083E+7	4.144E−3	4.573E−11
IL-27 to gp130-Fc	1.674E+11	4.563E+2	2.743E−9
Anti-gp130 to gp130-Fc	1.297E+4	1.070E−4	8.769E−9

Data are mean values of 3 independent experiments where each set of data was globally fitted using the 1:1 Langmuir binding model.

### The involvement of the MAPK/ERK and PI3K/AKT pathways

**FIG. 6.**
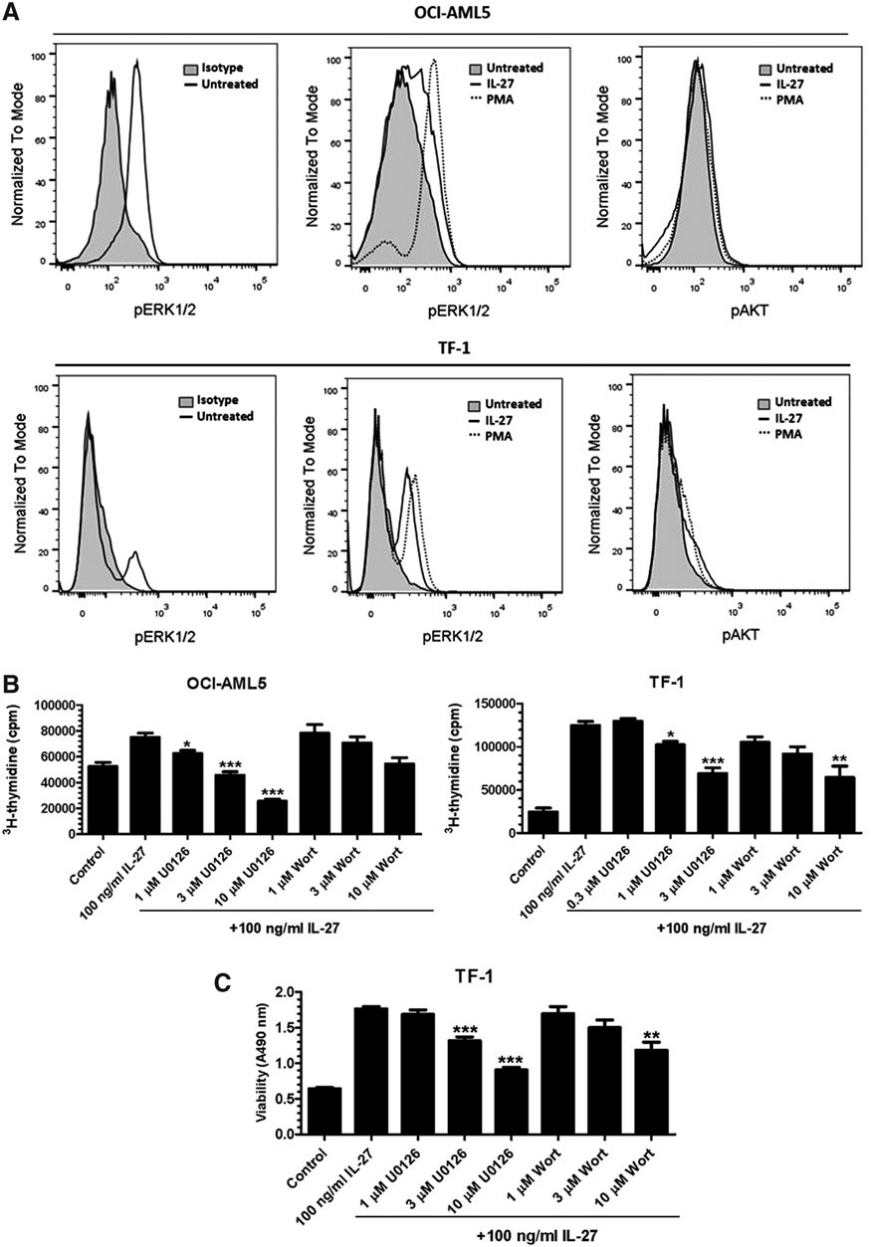
Activation of the MAPK/ERK and PI3K/AKT pathways is required for IL-27-mediated proliferation and survival. **(A)** Intracellular phosphorylation of ERK1/2-Thr202/Tyr204 and AKT-Ser47 by IL-27 was analyzed by flow cytometry on leukemic cell lines as indicated. **(B)** OCI-AML5 and TF-1 cells were deprived of GM-CSF and cultured in 10% or 5% serum assay medium containing IL-27 in the absence or presence of the MAPK/ERK inhibitor, U0126, or the PI3K/AKT inhibitor, wortmannin (Wort), at indicated concentrations. Cell proliferation was measured by ^3^H-thymidine incorporation after 2-day incubation. *P < 0.05, **P < 0.01 and ***P < 0.001 for IL-27 plus U0126 or Wort versus IL-27 alone. **(C)** TF-1 cells were deprived of GM-CSF and cultured in 2% serum assay medium containing IL-27 in the absence or presence of the MAPK/ERK inhibitor, U0126, or the PI3K/AKT inhibitor, wortmannin (Wort), at indicated concentrations. Cell viability was measured by formazan production after 48 h of incubation. **P < 0.01 and ***P < 0.001 for IL-27 plus U0126 or Wort versus IL-27 alone.

We further investigated the mechanisms by which IL-27 could favor leukemic cell proliferation and survival. The mitogen-activated protein kinase (MAPK)/ERK and phosphatidylinositol 3-kinase (PI3K)/AKT pathways play critical roles in transducing growth and survival signals from cell surface receptors activated by various cytokines (McCubrey and others 2008; Geest and Coffer 2009). The activation of ERK1/2 and AKT occurs through dual phosphorylation of Thr202/Tyr204 and single phosphorylation of Ser473, respectively. It has been demonstrated previously that IL-27 activated the MAPK/ERK cascade, which was required for Th1 differentiation (Owaki and others 2006). To test the hypothesis that IL-27 could promote cell proliferation and survival through activating the MAPK/ERK and PI3K/AKT pathways in leukemic cells, we examined the effects of IL-27 on intracellular phosphorylation of ERK1/2 at Thr202/Tyr204 and AKT at Ser473 by flow cytometry. We found that after serum deprivation of OCI-AML5 and TF-1 cells for 24 h, untreated controls still showed an increase in phosphorylation of ERK1/2 compared with isotype-matched controls, suggesting the constitutive activation of ERK1/2 to some extent in OCI-AML5 and TF-1 cells (Fig. 6A). As expected, the positive control PMA revealed a marked elevation of ERK1/2 phosphorylation compared with untreated controls. IL-27 treatment of OCI-AML5 and TF-1 cells also induced phosphorylation of ERK1/2, but only minimally affected AKT phosphorylation (Fig. 6A).

The involvement of the MAPK/ERK and PI3K/AKT pathways in IL-27-dependent cell growth and survival was further evaluated by using kinase inhibitors. Cells were pretreated with the specific mitogen-activated protein kinase (MEK) inhibitor, U0126, or the PI3K inhibitor, wortmannin, for 30 min in 10% serum medium, followed by stimulation with IL-27 for 2 days, and analyzed for cell proliferation. Pretreatment of OCI-AML5 cells with U0126 or wortmannin at various concentrations caused dose-dependent inhibition of IL-27-induced proliferation to the maximal level by 66% and 27%, respectively, compared with IL-27 alone (Fig. 6B). Similar to OCI-AML5 cells, IL-27-dependent growth of TF-1 cells was also attenuated by U0126 and wortmannin with 45% and 48% of inhibition, respectively (Fig. 6B). Furthermore, TF-1 cells were pretreated with or without the inhibitor U0126 or wortmannin in low serum assay medium and then incubated in the presence of IL-27 for 48 h, and the mitochondrial dehydrogenase activity measured. Consistent with observations on cell proliferation, U0126 or wortmannin treatment partially, but dose dependently, resulted in a reduction of the survival effects of IL-27 (Fig. 6C).

## Discussion

In the present study, we investigated the *in vitro* biological effects of IL-27 on IL-27R-expressing human leukemic cell lines. A major finding of this study is that IL-27 significantly stimulated proliferation of AML and erythroleukemic cell lines, including OCI-AML5, TF-1, UT-7, and UT-7/EPO cells. Consistent with this observation, IL-27 promoted cell survival of the leukemic cell lines. Our data are in agreement with previous studies (Pradhan and others 2007, 2010; Seita and others 2008; Rousseau and others 2010; Lambert and others 2011). Through functional screening approaches, Pradhan and others (2007, 2010) identified IL-27R as a transforming gene from the leukemic cells of a patient with AML and showed that IL-27R is highly expressed on the cell surface of bone marrow cells of AML patients compared with the normal bone marrow cells. IL-27R can transform hematopoietic cells through its ability to constitutively activate a mutant form of JAK2, suggesting that it may play unappreciated roles in myeloproliferative neoplasms. In support of this finding, Seita and others (2008) have shown that IL-27R is expressed on the surface of hematopoietic stem cells and aberrant expression of IL-27 induces proliferation of CD34^+^ hematopoietic stem cells and myelopoiesis in a transgenic mouse model. Another recent study by Lambert and others (2011) showed that mutations of IL-27R enhance the transforming activity of hematopoietic cells, suggesting the potential to contribute to hematopoietic neoplasms. Furthermore, on the basis of IL-27 structural analysis, Rousseau and others (2010) developed a mutated form of IL-27, which antagonized IL-27-induced proliferation of TF-1 cells.

Our reports of proliferative actions of IL-27 on leukemic cells conflict with other studies, which showed IL-27-mediated growth inhibition of primary leukemic cells from pediatric patients with B-acute lymphoblastic leukemia and AML (Canale and others 2011; Zorzoli and others 2012). The most likely explanation is due to differences in tumor cell subtypes, especially AML cells, given the heterogeneous nature of AML (Martelli and others 2013). Another possible explanation results from differences between childhood and adult leukemic cells as it has been reported that genetic abnormalities in pediatric AML differ from those of adults, highlighting the differences in AML biology over age groups (Creutzig and others 2012; Foran 2014). In addition, there were differences in experimental methodologies. Zorzoli and others (2012) reported antiproliferative activity of IL-27 on purified primary AML cells measured by intracellular staining Ki67, a nuclear marker for proliferation, whereas we determined growth stimulation of IL-27 as dose–response relationships of IL-27 with ^3^H-thymidine incorporation in leukemic cell lines. Finally, it is also possible that differences in potential dysregulation of expression and aberrant activation of IL-27R and its mediation of proliferative signals in cancer cells account for the opposing findings.

Another important finding of our study is that IL-27 desensitized OCI-AML5 cells to the clinically important chemotherapeutic agents, cytarabine and daunorubicin, as IL-27-treated OCI-AML5 cells were less susceptible to the cytotoxic activity of either cytarabine or daunorubicin compared with treatment with the chemotherapeutic alone. Clinically, AML is a common hematopoietic malignancy, and resistance to chemotherapy is one of the main reasons for recurrence and refractoriness of AML. Therefore, it is very important to understand the mechanisms involved that may lead to improved treatment. Consistently, the decreased chemosensitivity by IL-27 was also observed in the other IL-27-responsive erythroleukemic cell lines, TF-1, UT-7, and UT-7/EPO. The ability of IL-27 to affect drug sensitivity to leukemic cells has not been previously studied. Our data suggest that IL-27 may play a role in regulation of chemoresponse of leukemic cells. Further studies are required to explore the mechanisms by which IL-27 mediates chemoresistance.

The ability of IL-27 to regulate cell apoptosis was assessed by measuring both Annexin V binding to the exposed phosphatidylserine and the activity of caspases 3 and 7. Our study demonstrated that IL-27 did not cause apoptosis as there was no increase in both Annexin V+ cell population and caspase-3/7 activity in the treated cells. Interestingly, IL-27 decreased both TNF-α-induced apoptotic Annexin V+ cells and the activity of caspase-3/7. The IL-27-mediated antiapoptosis is consistent with its growth stimulation activity in leukemic cells. The mechanism of IL-27 antagonism of TNF-α-induced apoptosis in leukemic cells is unclear. It has previously been reported that IL-27 suppressed responses of macrophages to TNF-α through downregulating cell surface expression of the TNF-α receptors, p55 and p75 (Kalliolias and others 2010). Thus, it is conceivable that IL-27 may use similar mechanisms to induce antiapoptotic activity through modulating TNF-α receptor expression.

A novel finding of the present study is that IL-27 can interact directly with each chain of IL-27R, either IL-27Rα or gp130, with markedly differential binding kinetic profiles and affinities, which may be correlated with biological activities mediated by each receptor chain. We showed that both IL-27-dependent STAT1/3 activation and cell proliferation were affected by blocking the IL-27/IL-27R system using either the IL-27Rα-Fc fusion protein or the neutralizing anti-gp130 Ab, but the extent of inhibition varied. Furthermore, addition of the IL-27Rα-Fc fusion protein and anti-gp130 Ab together completely blocked IL-27-induced proliferation, which was in agreement with previous studies demonstrating that both chains of IL-27R are required for mediating maximal cellular responses to IL-27 (Pflanz and others 2004). However, the difference observed in the antagonism of IL-27 function indicates different biological contributions mediated by each receptor subunit in response to its ligand. Additionally, the gp130-Fc fusion protein failed to show any effect on IL-27-induced STAT1/3 activation. This finding was consistent with previous data showing that a recombinant IL-27Rα-Fc fusion protein acted as an inhibitor of IL-27 activity, whereas a recombinant soluble gp130-Fc fusion protein had no effect on IL-27-induced STAT1/3 activation (Scheller and others 2005; Wirtz and others 2006). These observations prompted us to investigate the relationship of binding characteristics of IL-27 to its receptor component with its biological actions. SPR revealed strikingly differential binding kinetic profiles of IL-27 to each receptor chain. IL-27 exhibited much higher binding affinity for IL-27Rα than for gp130, which may explain why the gp130Fc protein was not potent enough to affect IL-27 binding to IL-27Rα on the cell surface to initiate subsequent STAT signaling events. The fact that the blockade of IL-27/IL-27Rα was more effective than the antagonism of IL-27/gp130 strongly suggests that the signaling and biological effects of IL-27 on leukemic cells are mediated largely through the IL-27Rα subunit and to a lesser extent through the gp130 subunit. Mechanistically, these results have implications for understanding how IL-27 interaction with each receptor chain makes different contributions to the biological outcomes.

To elucidate the molecular mechanisms underlying IL-27 signaling pathways leading to cell proliferation and survival, we investigated the involvement of the MAPK/ERK and PI3K/AKT signaling events. We showed that IL-27 induced phosphorylation and activation of ERK1/2 in leukemic OCI-AML5 and TF-1 cells, and the specific inhibitor of MAPK/ERK (U0126) strongly decreased the IL-27-dependent proliferation of OCI-AML5 and TF-1 cells. This agrees with previous reports, which showed that IL-27 induces cell proliferation through the same MAPK/ERK-dependent pathway (Owaki and others 2006). However, the PI3K/AKT inhibitor (wortmannin) caused a significant reduction of IL-27-mediated proliferation only in TF-1 cells, but not in OCI-AML-5 cells, suggesting that the involvement of the PI3K/AKT pathway appears to be cell line dependent. Moreover, TF-1 leukemic cell survival induced by IL-27 was more effectively reduced by the MAPK/ERK inhibitor than the PI3K/AKT inhibitor. Thus, our findings suggest that the MAPK/ERK signaling cascade is primarily required for IL-27-induced tumor growth and survival.

In conclusion, we have demonstrated for the first time that IL-27 can regulate tumor cell growth by favoring proliferation of human AML and erythroleukemic cells, suggesting that IL-27 may represent a promising potential therapeutic target for certain subtypes of leukemia. We have also shown that IL-27 plays a role in modulating chemosensitivity to cytarabine and daunorubicin, suggesting that IL-27 may be an important determinant of drug resistance in AML and erythroleukemic cells. Our data together with previous reports indicate that IL-27, like its distinct properties in immune responses, may either stimulate or inhibit cancer cell growth depending on the context of different subtypes of leukemia. These findings extend our knowledge on the diverse biological activities of IL-27 and the complex nature of its effects on certain subtypes of human leukemia.
